# Combined treatment with electrical stimulation and insulin-like growth factor-1 promotes bone regeneration in vitro

**DOI:** 10.1371/journal.pone.0197006

**Published:** 2018-05-10

**Authors:** Zhiping Qi, Peng Xia, Su Pan, Shuang Zheng, Chuan Fu, Yuxin Chang, Yue Ma, Jincheng Wang, Xiaoyu Yang

**Affiliations:** 1 Department of Orthopedic Surgery, the Second Hospital of Jilin University, Changchun, PR China; 2 Department of Gynecological Oncology, the First Hospital of Jilin University, Changchun, PR China; Kyungpook National University School of Medicine, REPUBLIC OF KOREA

## Abstract

Electrical stimulation (ES) and insulin-like growth factor-1 (IGF-1) are widely used in bone regeneration because of their osteogenic activity. However, the combined effects of ES and supplemental IGF-1 on the whole bone formation process remain unclear. In this study, fluorescence staining and an MTT assay were first utilized to observe the influence of ES and IGF-1 on MC3T3-E1 cell proliferation and adhesion in vitro. Subsequently, osteogenic differentiation was evaluated by the alkaline phosphatase activity (ALP) and the expression of osteogenic marker genes. In addition, cell mineralization was determined by alizarin red staining and scanning electron microscopy (SEM). We demonstrated that the MC3T3-E1 cell proliferation was significantly higher for treatments combining IGF-1 and ES than for treatments with IGF-1 alone. The combination of IGF-1 and ES increased the MC3T3-E1 cell ALP activity, the expression of osteogenesis-related genes and the calcium deposition with a clear dose-dependent effect. Our data show the synergistic effect of IGF-1 and ES in promoting the proliferation, differentiation and mineralization of MC3T3-E1 cells, which suggests that it would be more effective to combine the proper dose of IGF-1 with ES to promote local bone damage repair and regeneration.

## Introduction

Further intervention may end in serious morbidities, such as increased pain and functional limitations, due to nonfulfillment or a slowdown in bone healing[1]. The end purpose of the treatment of patients with fractures and surgical osteotomies is a compound physiological process in bone healing. Across a range of symptoms, electrical stimulation (ES) is a widely known adjunctive therapy used to enhance bone healing[2–5]. A recent randomized sham-controlled trial to determine the effect of electrical stimulation on bone healing has been performed through meta-analysis. This study found that patients treated with electrical stimulation have significantly less pain and experience lower rates of radiographic nonunion or persistent nonunion[6]. In addition, the correlations between ES and osteogenic proliferation and differentiation have been detailed[5, 7]. ES could influence bone marrow mesenchymal stem cell (BMMSC) activities, and degenerate wave and capacitive coupling can increase cell proliferation[8]. Pulsed DC ES can modify the higher expression of osteogenic marker genes, including RUNX2, osteopontin (OPN) and osteocalcin (OCN) in BMMSCs[9]. Wiesmann H et al. showed that osteoblasts are sensitive to ES, which enhances the mineralization process[10]. All of these results, based on clinical evidence and the scientific research on bone healing, revealed that electric stimulation can genuinely ameliorate osteogenesis.

In addition, the biophysical signals triggered by ES are used for a wide range of bone disorders, and various of growth factors, such as bone morphogenetic proteins (BMPs), epidermal growth factor (EGF) and insulin-like growth factor-1 (IGF-1) have also been implicated in osteogenesis[11–14]. Evidence indicates that IGF-I is one of the most abundant growth factors in the bone matrix, which influences a wide range of physiological functions, including growth, differentiation and metabolism[15]. IGF-1 has attracted attention as a therapeutic factor in fracture healing, either alone or in a combined treatment, due to its positive effect on osteogenesis[16–18]. A previous study showed the IGF-1 knockout mice had an obvious reduction in bone formation rates and hypomineralization of the skeleton[19]. In contrast, transgenic mice with overexpression of IGF-1 had better cortical and trabecular bone growth and mineralization[20, 21]. Furthermore, it has been established that IGF-1 plays a critical role in the whole bone formation process, although the proper dose for osteoblast proliferation, differentiation and mineralization has been debated[15, 22, 23].

Previous studies showed that IGF-1 is a key marker of physiological fracture healing and osteoblast maturation, and IGF-1 was proved to have a synergistic effect with other factors, such as BMP-2 and BMP-6, in a combination treatment[23, 24]. Because IGF-1 is a structurally simpler and significantly cheaper growth factor than the traditional BMPs, and because ES is a flexible, safe, and cheap way to treat bone fracture and nonunions, we wondered whether an IGF-1 and ES combined treatment could be a new strategy for bone healing. Even though the IGF-1 and ES combination therapy was proved to have a synergistic effect on cardiac development[25], their function on bone regeneration remained unclear. Therefore, we evaluated the cell proliferation and adhesion, the mRNA level of osteosis-related genes (RUNX2, OPN and COL I), markers of osteogenesis differentiation (ALP activity), and intracellular calcium deposition. The present study aimed to demonstrate whether IGF-1 alone or combined with ES was able to regulate the functions of osteogenesis, as well as the appropriate doses.

## Materials and methods

### Cell culture

The osteoblastic cell line MC3T3-E1 has been established from a C57BL/6 mouse calvaria and has the capacity to differentiate into osteoblasts and osteocytes in vitro. In our study, MC3T3-E1 cells were obtained from the Cell Culture Center of Institute of Basic Medical Sciences Chinese Academy of Medical Sciences (Shanghai, China). MC3T3-E1 cells were cultured and expanded in basal medium containing Dulbecco’s modified Eagle’s medium (DMEM) supplemented with 10% fetal bovine serum (Gibco, USA), 100 units/ml penicillin (Sigma), and 100 mg/ml streptomycin (Gibco, USA). Cells at passage 2–3 were grown in a 24-well cell culture plate (Corning, USA) at initial seeding cell densities of 20,000 cells/well under a controlled temperature condition (37°C) with a humidified atmosphere of 95% air and CO_2_ (5%). The medium was replaced with fresh medium every 2 days.

### Electrical stimulation

**Fig 1:**
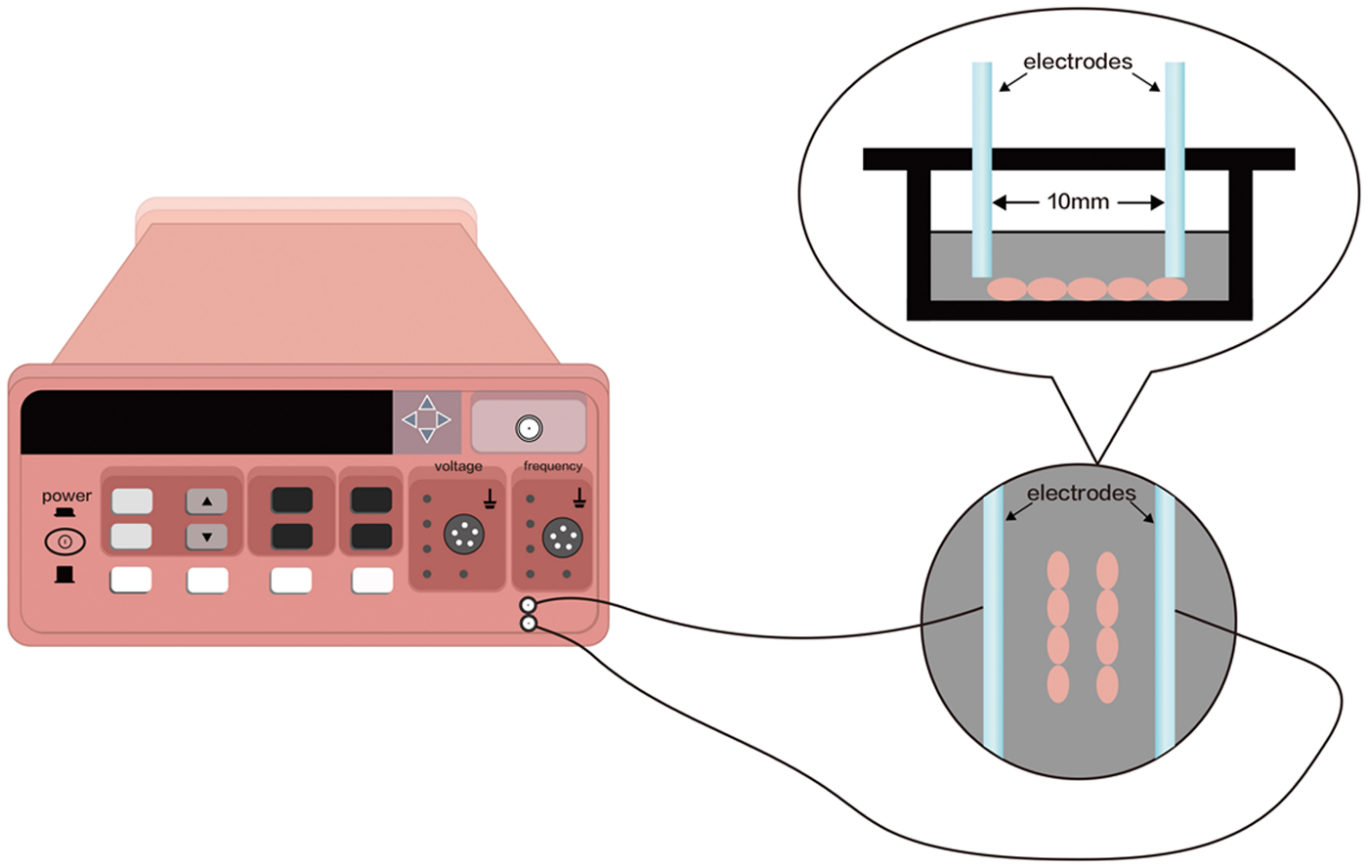
Set-up of electrical stimulation. L-shaped platinum electrodes are connecting to a power supply and delivering electrical stimulation to the cells.

The set-up of the ES was shown in Fig 1. To produce a uniform electric field in the culture medium, a pair of L-shaped platinum electrodes separated by a distance of 10 mm was placed in the lid of 24-well cell culture plates. For sterilization, the electrodes were submerged in 70% ethanol for 10 min, washed with sterile calcium-magnesium free phosphate buffer saline (PBS) and finally exposed to UV light overnight. A function signal generator (Suing, China) was connected to the electrodes via alligator clips and copper wires to create a signal source. Output signals from the signal generator were monitored by a digital oscilloscope. MC3T3-E1 cells that received electrical stimulation were exposed to 100 mV of ES for 1 h/day. The ES frequency was programmed at 50 Hz to 800 Hz, with a stable 50% duty cycle and rectangular pulses. All evaluations and assays were performed 24 h after the last exposure.

### Cell proliferation and adhesion assays

Cell proliferation was assessed by a (4,5-dimethylthiazol-2-yl)-2,5-diphenyltetrazolium bromide (MTT) assay (Sigma-Aldrich, USA) for 1, 3 and 7 days. First, 100 μL of MTT (Sigma) stock solution in PBS (5 mg/mL) was added to each well, and the cells were incubated at 37°C for another 4 h. Then, the medium was removed, and 750 μL of acidified isopropanol (2 mL of 0.04 N hydrochloric acid (HCl) in 100 mL of isopropanol) was added to each well to dissolve the formazan crystals. The optical density (OD) was measured at a 540 nm wavelength on a Full Wavelength Microplate Reader (Infinite M200, TECAN). For the cell adhesion examination, the cell samples were rinsed twice with PBS and fixed with 4% paraformaldehyde for 30 min at room temperature. Actin filaments were stained by incubating the samples with rhodamine phalloidin (Invitrogen, Eugene, OR, USA), and the cell nucleus was stained using 4′,6-diamidino-2-phenylindole (DAPI; Sigma-Aldrich, USA). The MC3T3-E1 morphology was observed under a fluorescence microscope (TE2000-U, Nikon).

### Quantitative real-time PCR analysis

The expression of osteogenic marker genes, RUNX2, collagen I (Col I), and OPN, was analyzed by real-time polymerase chain reaction (RT-PCR). The total RNA concentration and purity were determined by a Nanodrop Assay (Tecan M200). The first strand cDNA was synthesized by reverse transcriptase as described in the M-MLV manual (Promega). Gene-specific primers were designed using the primer design software Beacon 5.0. The specificity of oligonucleotides was checked by BLASTN^®^ (Basic Local Alignment Search Tool) against the mouse RefSeq RNA database at NCBI. All samples were assayed in triplicate in 8 striped optical tubes (Axygen) using a qPCR SYBR Green Mix Kit (Stratagene). The PCR amplification was performed as follows: initial heating at 95°C for 10 min, followed by 40 cycles at 95°C for 30 s, 58°C for 60 s, and 72°C for 60 s. Each gene expression value was normalized to that of the housekeeping gene, glyceraldehyde-3-phosphate dehydrogenase (GAPDH). The results are reported as the relative gene expression. Information on the primers is provided in Table 1.

**Table 1:** List of genes and the primer nucleotide sequences.

Gene	Forward primer sequence	Reverse primer sequence
RUNX2	5-GCCCTCATCCTTCACTCCAAG-3′	5-GGTCAGTCAGTGCCTTTCCTC-3′
OPN	5-TCAGGACAACAACGGAAAGGG-3′	5-GGAACTTGCTTGACTATCGATCAC-3′
COL I	5-CGCTGGCAAGAATGGCGATC-3′	5-ATGCCTCTGTCACCTTGTTCG-3′
GAPDH	5-AATGTGTCCGTCGTGGATCTG-3′	5-CAACCTGGTCCTCAGTGTAGC-3′

### Immunofluorescence staining

MC3T3-E1 cells cultured for 7 days were fixed with 4% paraformaldehyde in PBS for 20 min. The samples were permeabilized with 0.1% Triton-X 100 in phosphate buffer for 5 min. After being blocked using 1% BSA in phosphate buffer for 30 min, the samples were incubated with primary antibody (1:500, Abcam) for 60 min at room temperature. Subsequently, the samples were washed by PBS three times, followed by incubation in fluorescein isothiocyanate (FITC)-labeled secondary antibody (1:500, Abcam) at ambient temperature in the dark for 60 min. Finally, the cell nuclei were dyed with 4′,6-diamidino-2-phenylindole (DAPI) for 1 min. Photos were taken on a confocal laser scanning microscope (LSM 780, ZEISS).

### ALP assay

After culturing for 7 and 14 days in various conditions, the cells were assayed for ALP activity with a p-Nitrophenyl Phosphate (pNPP) Liquid Substrate System (Sigma) following the manufacturer’s instructions. For morphological observation, the cells were stained using an alkaline dye mixture of 0.01% (W/V) Naphthol AS-MX Phosphate Alkaline Solution and 0.24 mg/ml Fast Blue RR salt at 18–26°C for 30 min. After 30 min Mayer’s Hematoxylin Solution was used for staining for 10 min. For quantitative determination, cells were first lysed in lysis buffer (Sangon) for 30 min and then transferred to a 96-well transparent plate. Next, pNPP solution was added to each well, and the plate was incubated in the dark for 30 min at 37°C. The plate was read at 405 nm on a multifunction microplate scanner (Infinite M200, TECAN).

### Mineralization

Calcium deposition was determined by alizarin red S (ARS) staining of the MC3T3-E1 cells. The cells were first fixed in 4% paraformaldehyde in PBS for 20 min and then washed with acidic PBS (pH 4.2) three times. Then, ARS (50 mM) was used to stain the cells for 20 min at room temperature, followed by treatment with 1 mL of 10% CPC solution. The absorbance of the solution was read at 540 nm by a multifunctional microplate scanner. The samples were stained with alizarin red S solution (50 mM) for 20 min at 37°C. Furthermore, the cell samples cultured for 20 days were fixed with 4% glutaraldehyde solution and dehydrated in graded ethanol solutions (50, 70, 80, 90, 95 and 100%). The samples were coated with gold and observed by SEM.

### Statistical analysis

All quantitative data were analyzed with OriginPro 8.0 (Origin Lab Corporation, USA) and presented as the mean ± standard deviation. Statistical differences were assessed by one-way analysis of variance (ANOVA). A value of p < 0.05 was considered to be significant.

## Results

### Cell proliferation under ES with different frequency

**Fig 2:**
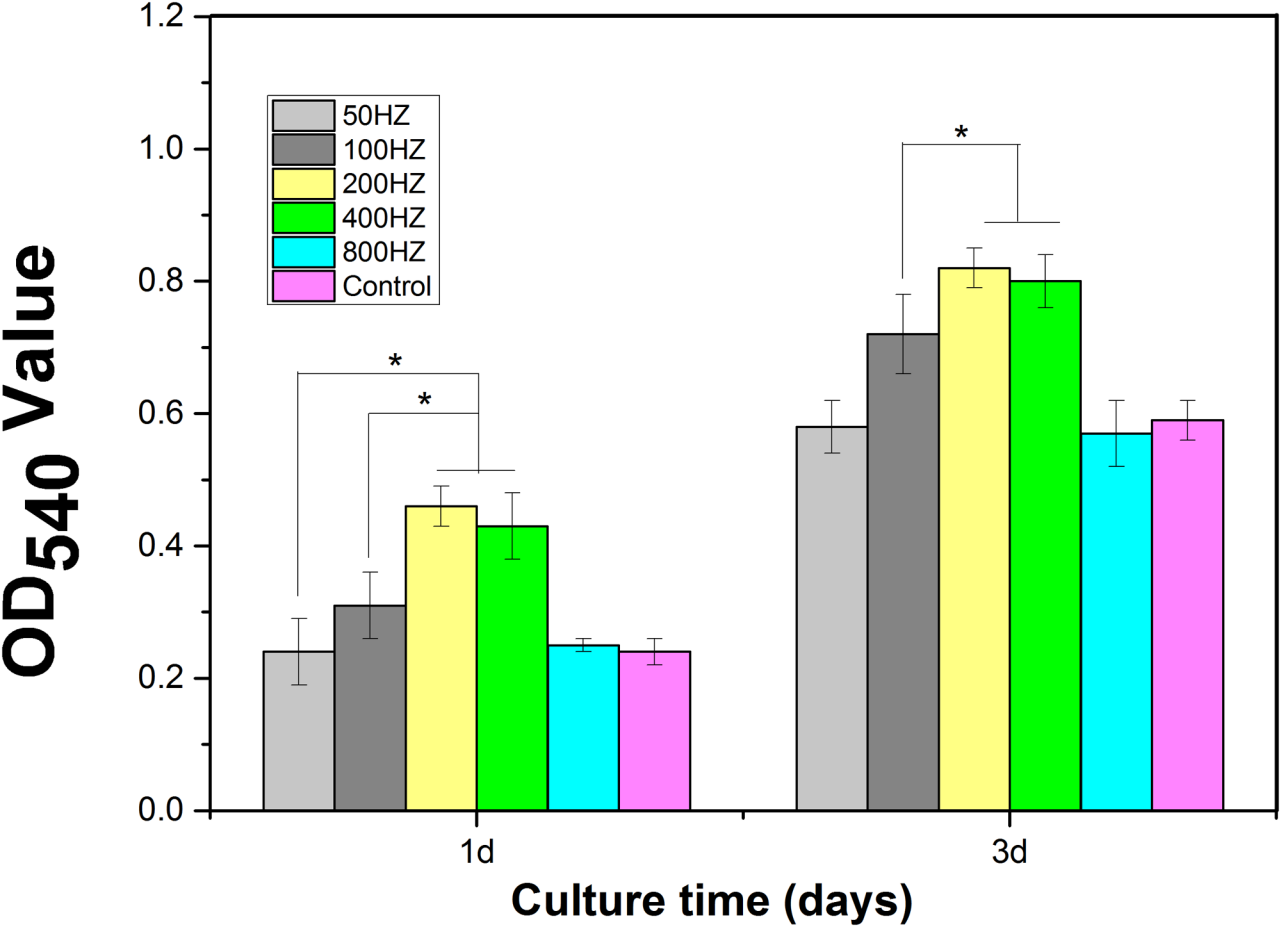
Proliferation of MC3T3-E1 cells cultured under ES with a different frequency for 1 and 3 days in vitro. (*, p < 0.05, n = 4).

To examine the appropriate ES frequency for further experiments, we used an MTT assay to evaluate the cell proliferation. As shown in Fig 2, the metabolic activity (OD value) of the MC3T3-E1 cells of all three groups increased gradually with culture time. There was no significant difference among the 50 Hz, 800 Hz and control groups at 1 and 3 d (p > 0.05). After 1 d and 3 d of culture, the cell numbers of the 200 Hz and 400 Hz groups were significantly higher than those of the other ES groups (p < 0.05). We found that the 200 Hz group had the maximum OD value compared to those of the other groups, although there were no significant differences between the 200 Hz and 400 Hz group (p > 0.05). Therefore, we choose the frequency of 200 Hz for the subsequent experiments.

### Cell proliferation and adhesion after combined treatment with IGF and ES

**Fig 3:**
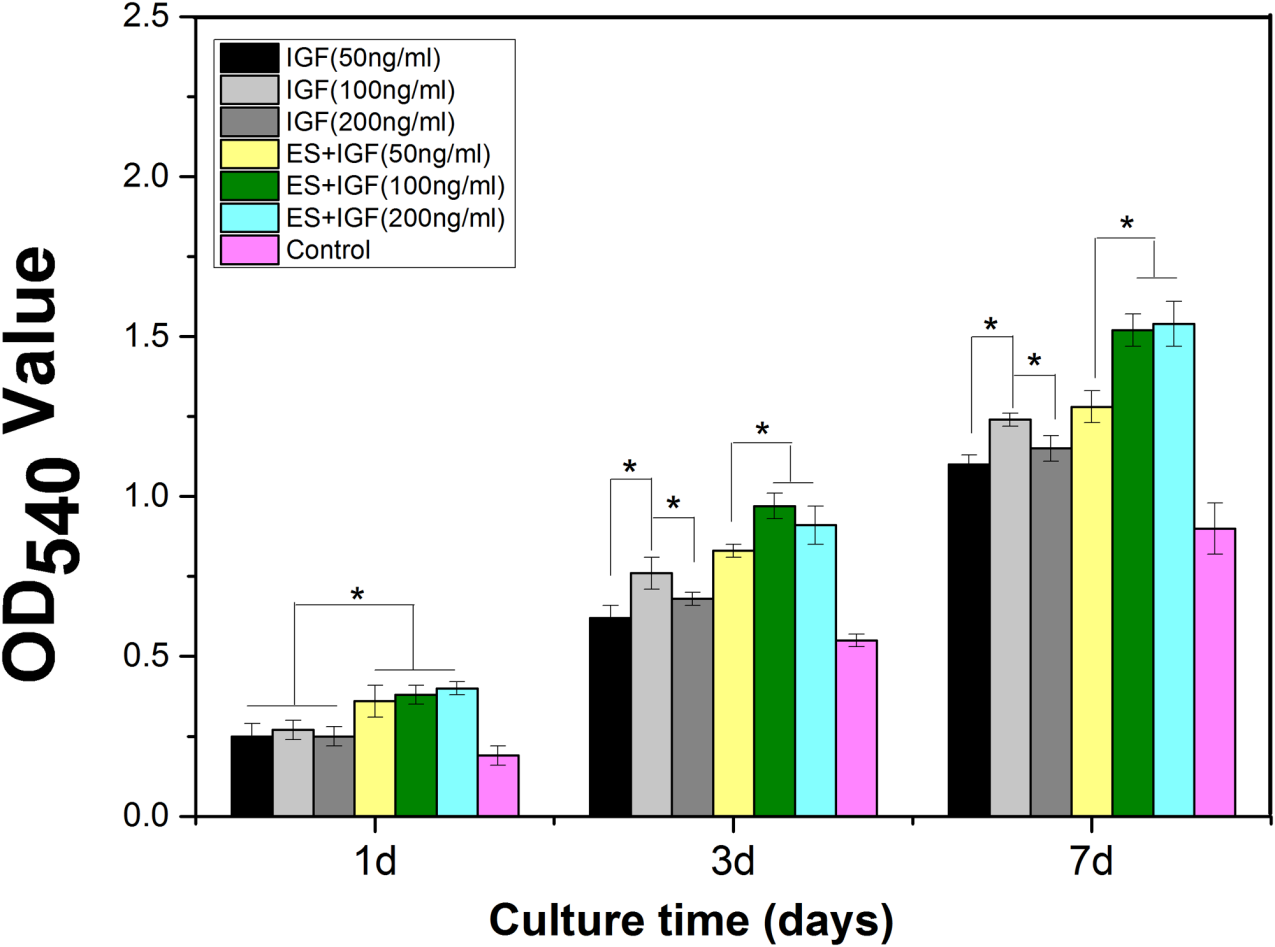
Proliferation of MC3T3-E1 cells cultured under IGF + ES combined treatment for 1, 3, 7 days in vitro. (*, p < 0.05, n = 4).

**Fig 4:**
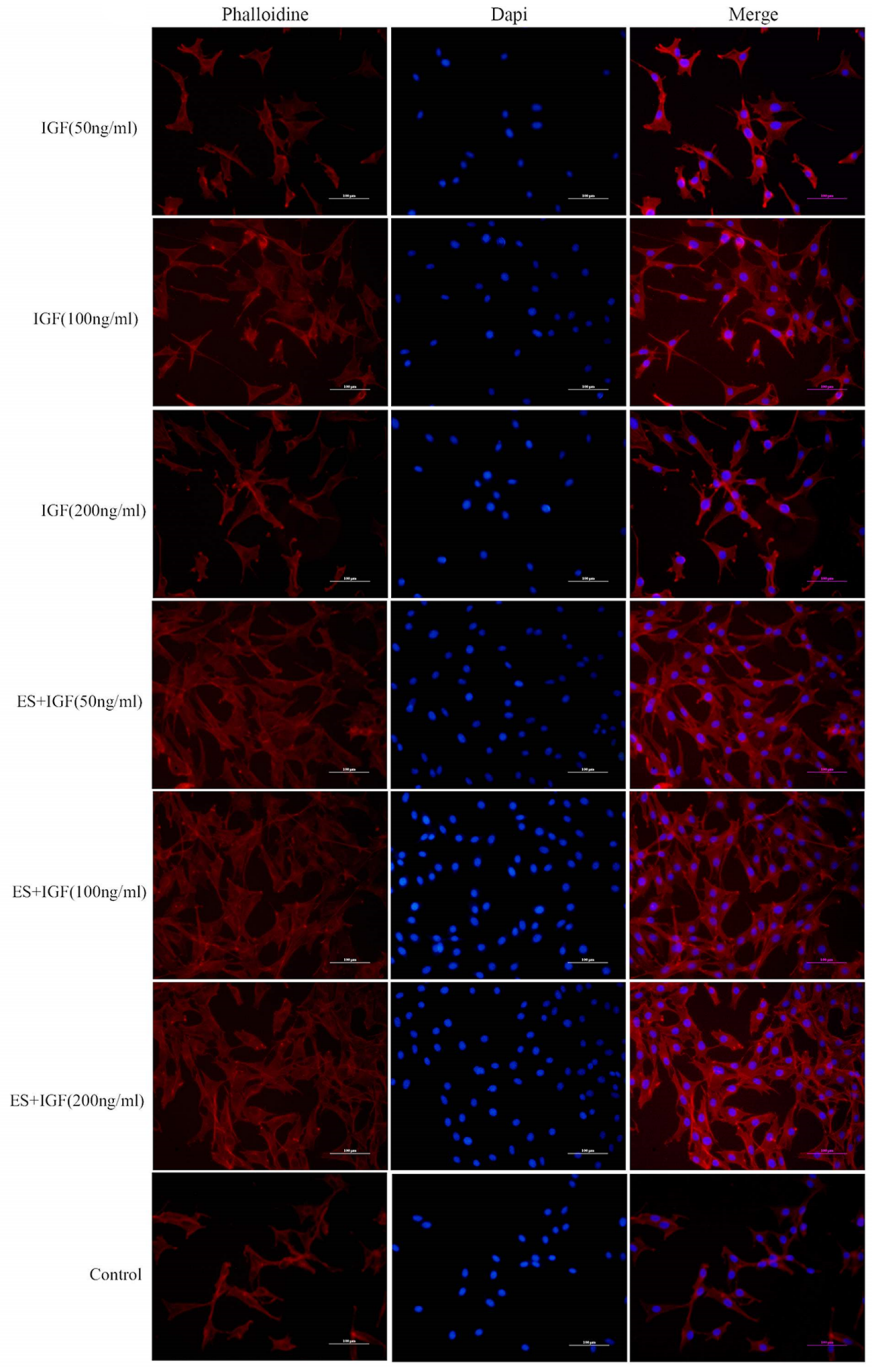
Morphology of MC3T3-E1 cells cultured under the ES+IGF combined treatment for 1 day in vitro. All scale bar lengths are 100 μm.

To investigate the effect of IGF and ES individually and together on cell proliferation and adhesion, an MTT assay was performed, and the morphology of the MC3T3-E1 cells at 1 day was investigated. The cell proliferation was significantly greater in the ES+IGF group than in the IGF alone groups at the same concentration at 1, 3 and 7 days (p < 0.05, Fig 3). We found that the OD value was significantly higher in the three IGF alone groups than in the control group, while 100 ng/mL IGF was most effective in the enhancement of cell proliferation among the three concentrations used in this study at 3 and 7 days, although there was no significant difference among the three IGF groups at 1 day. Furthermore, a significant difference in cell proliferation was observed between ES+IGF (100 ng/ml) and the other two ES+IGF groups at 3 and 7 days. We used actin fluorescence staining of microfilaments (red) and nuclei (blue) to observe the cell morphology. As shown in Fig 4, at 1 day post-seed, the cytoskeletal and nuclear staining showed that the MC3T3-E1 cells cultured in ES+IGF exhibited a greater spread with a better cytoskeleton than that of the IGF alone and control groups. The control group and the cells grown with IGF alone were shrunken and had fewer pseudopods around the cells.

### Quantification of gene expression

**Fig 5:**
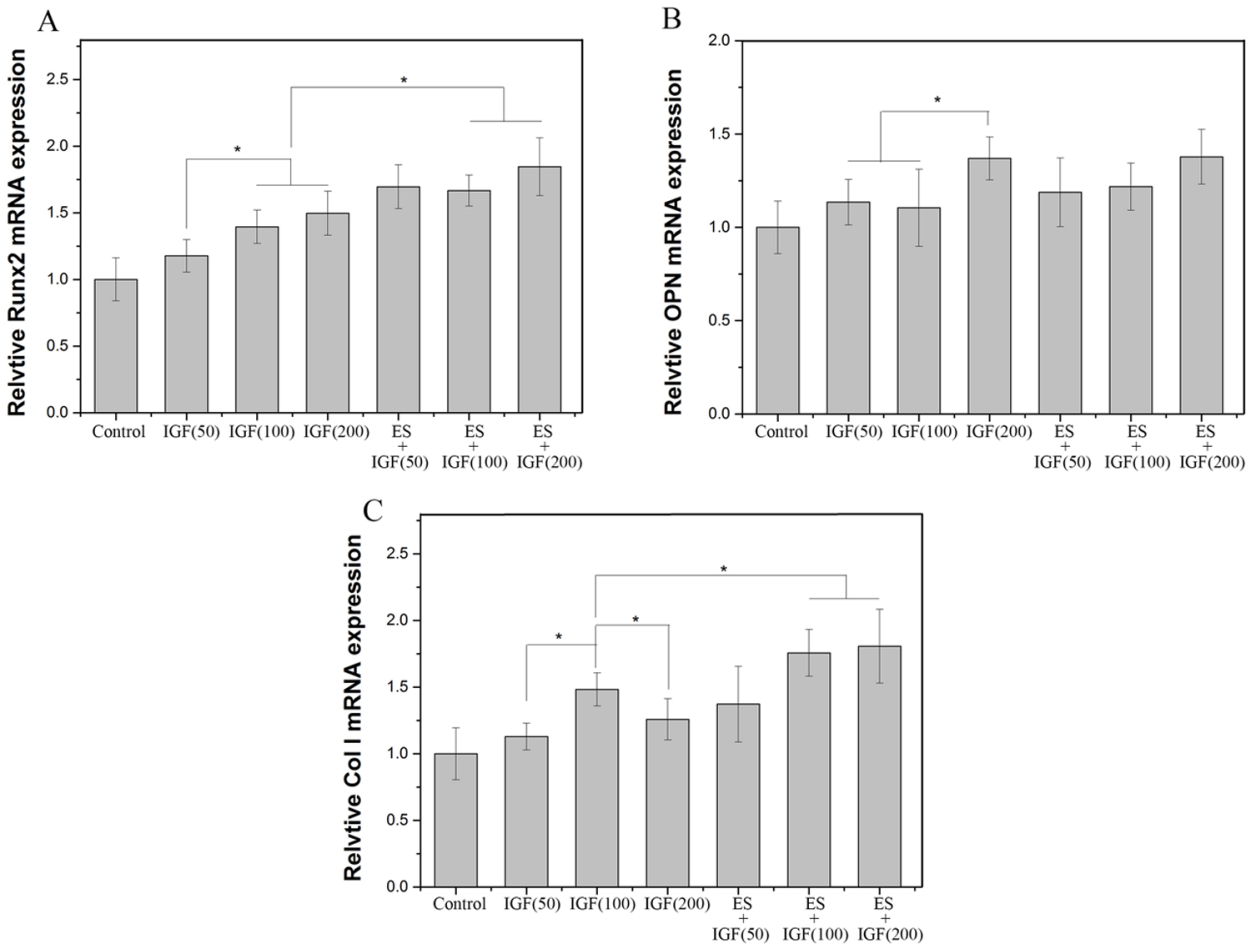
Quantitative real-time PCR analysis of the osteogenesis-related gene expression of RUNX2 (A), OPN (B) and Col I (C) after MC3T3-E1 cells were cultured for 7 days. (*, p < 0.05, n = 4).

**Fig 6:**
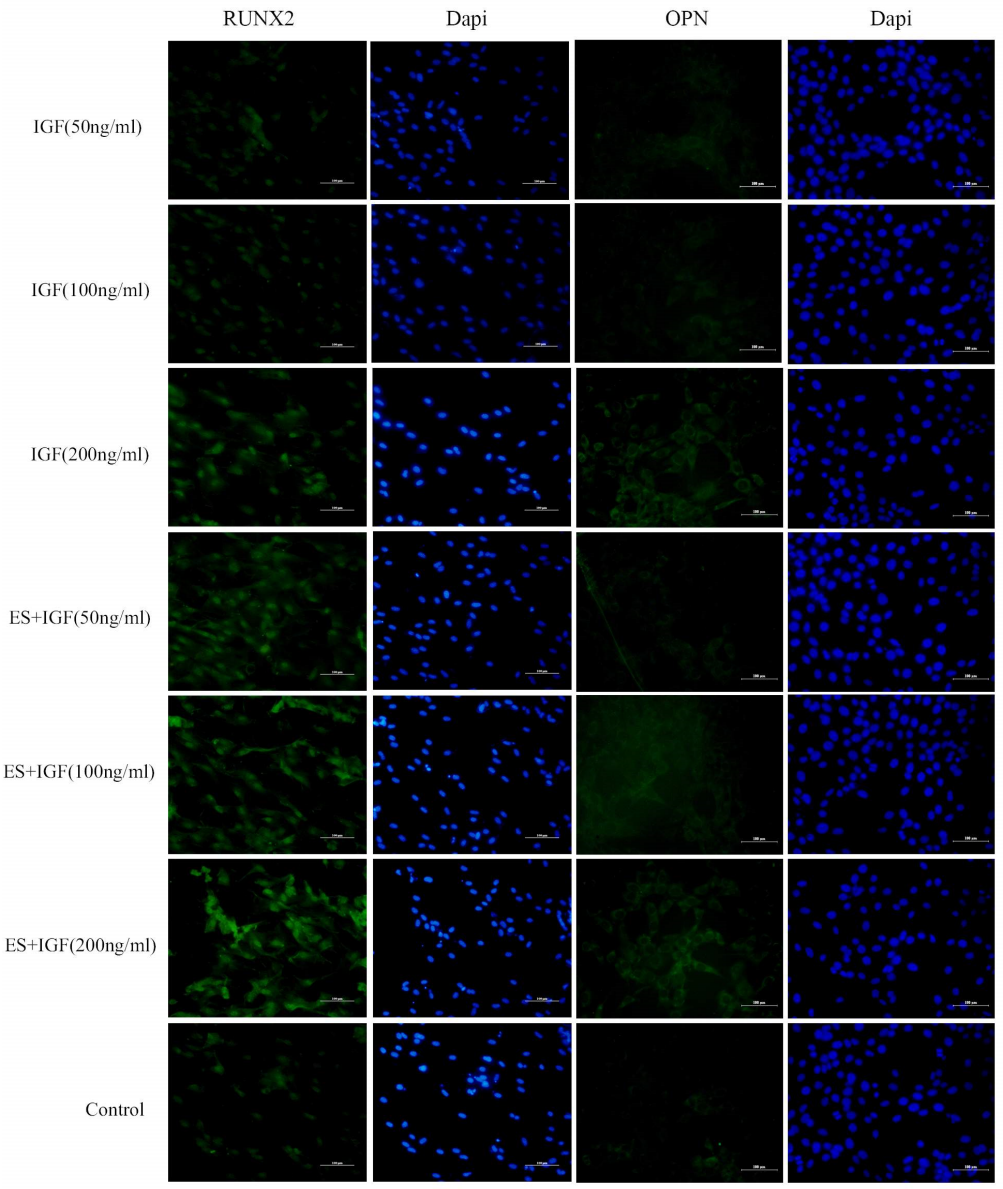
In vitro RUNX2 and OPN protein expression of MC3T3-E1 cells cultured for 7 d. All scale bar lengths are 100 μm.

RUNX2, OPN and Col I are three key osteoblast markers in the process of osteogenic differentiation. The expression of the osteogenesis-related genes during differentiation at 7 days was quantitatively analyzed using qRT-PCR. The mRNA level of RUNX2 gradually increased as the IGF dose increased and reached the maximum when the concentration of IGF-1 was 200 ng/ml. Significantly higher RUNX2 gene expression was observed in the ES+IGF groups than in the IGF alone groups at the same concentration and the control group (p < 0.05, Fig 5). The OPN expression in the 200 ng/ml IGF group was significantly higher than that in the other IGF alone groups, while there was no significant difference between ES+IGF groups and IGF alone groups at same concentration (p > 0.05). An obvious increase in Col I mRNA was observed in the 100 ng/ml IGF group compared with the three IGF groups. Furthermore, the ES+IGF (100 ng/ml) and ES+IGF (200 ng/ml) groups had dramatically higher Col I expression than the IGF (100 ng/ml) and IGF (200 ng/ml) groups respectively. The findings of this study showed that 100–200 ng/ml IGF can enhance the osteodifferentiation of MC3T3-E1 cells, especially their RUNX2 and Col I gene expression. To better observe cell osteogenesis differentiation, the protein expression of RUNX2 and OPN of the MC3T3-E1 cells was evaluated by immunofluorescence staining. As shown in Fig 6, after 7 days of culture, the RUNX2 protein expression was higher in the ES+IGF groups than in the IGF alone groups, while OPN was expressed when the IGF concentration was 200 ng/ml.

### ALP activity

**Fig 7:**
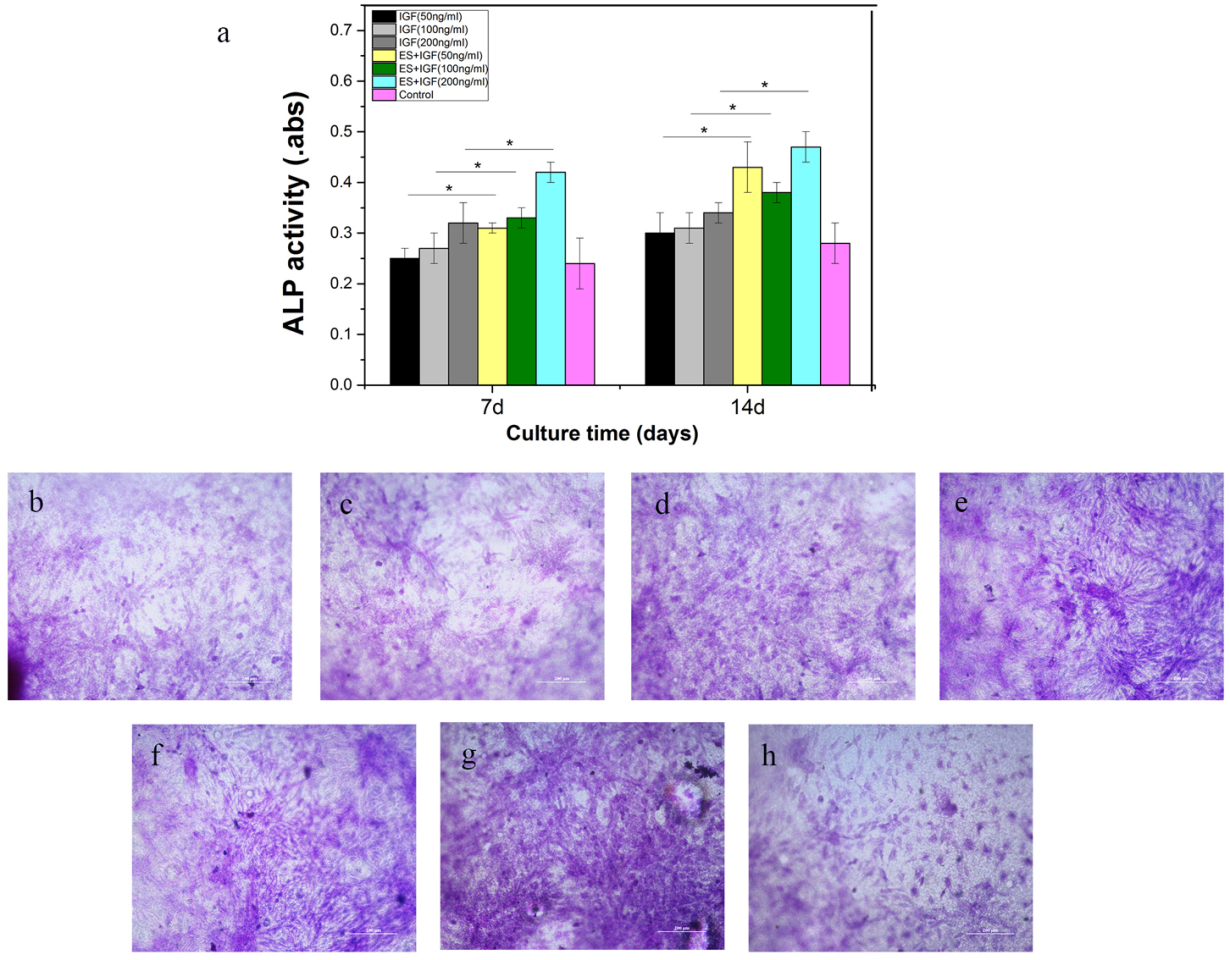
(a) ALP activity quantitative analysis by a pNPP assay at 7 d and 14 d. (*, p < 0.05, n = 4). ALP activity staining in the (b) IGF (50 ng/ml), (c) IGF (100 ng/ml), (d) IGF (200 ng/ml), (e) ES+IGF (50 ng/ml), (f) ES+IGF (100 ng/ml), (g) ES+IGF (200 ng/ml), and (h) control groups at 14 days. All scale bar lengths are 200 μm.

ALP is an early marker of osteogenic differentiation, and the ALP activity in MC3T3-E1 cells was examined to evaluate the osteoinductive activity. An obvious increase in the ALP reactivity of MC3T3-E1 cells was found in the ES+IGF groups compared with the IGF alone groups at both 7 and 14 days (p < 0.05, Fig 7a). The ALP activity of the IGF alone groups gradually increased as the IGF concentration increased and reached a maximum when the IGF concentration was 200 ng/ml at both 7 and 14 days. The ALP activity was significantly greater in the ES+IGF groups than in the IGF alone groups with the same concentration at 7 and 14 days. Cells were histochemically stained for ALP activity to assess the osteogenic differentiation (Fig 7b–7h). The ALP activity increased when IGF was administered with ES.

#### Mineralization

**Fig 8:**
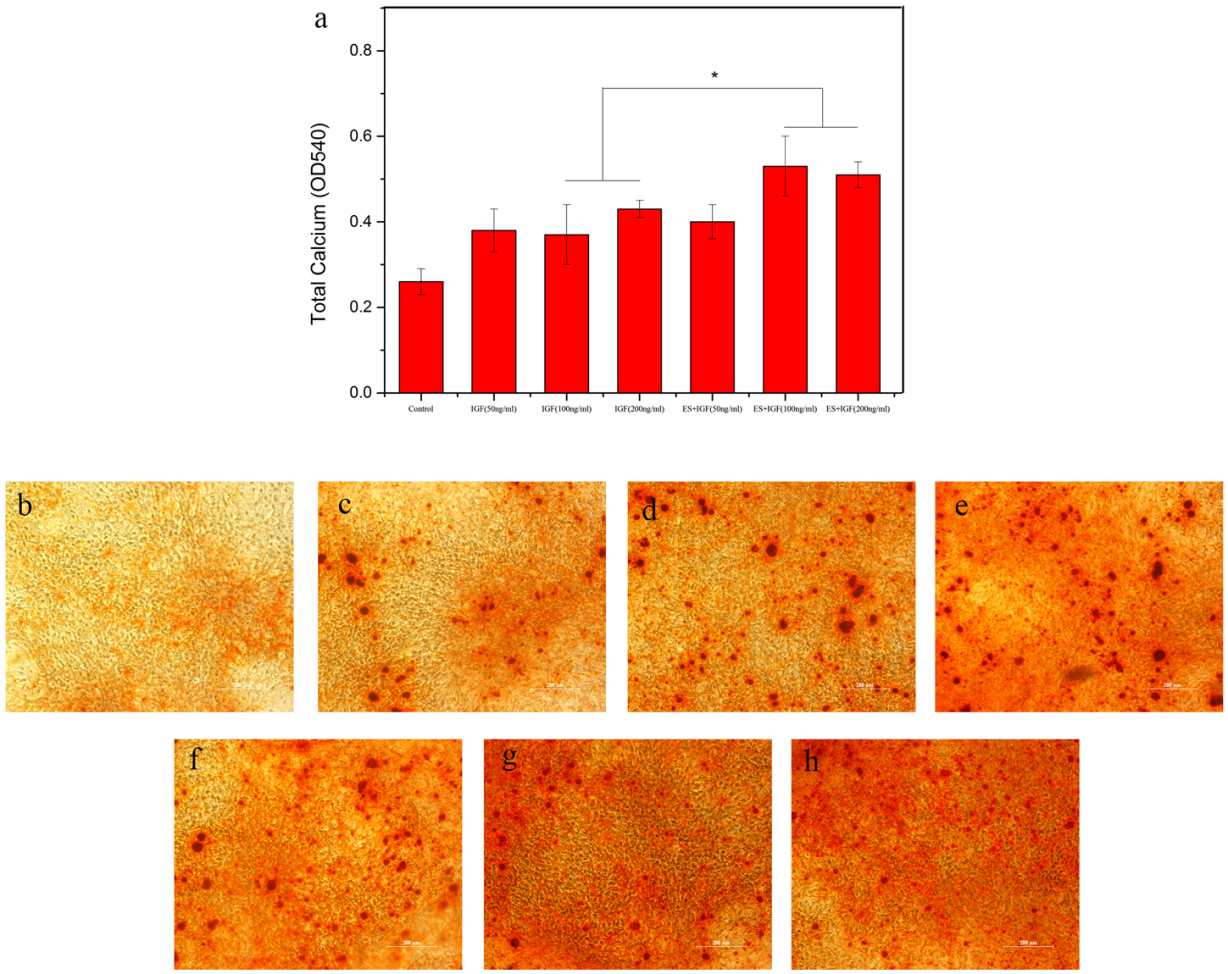
(a); Calcium content mineral deposition determined by ARS analysis at 21 d. (*, p < 0.05, n = 4). ARS staining in the (b) control, (c) IGF (50 ng/ml), (d) IGF (100 ng/ml), (e) IGF (200 ng/ml), (f) ES+IGF (50 ng/ml), (g) ES+IGF (100 ng/ml), and (h) ES+IGF (200 ng/ml) groups at 21 days. All scale bar lengths are 200 μm.

**Fig 9:**
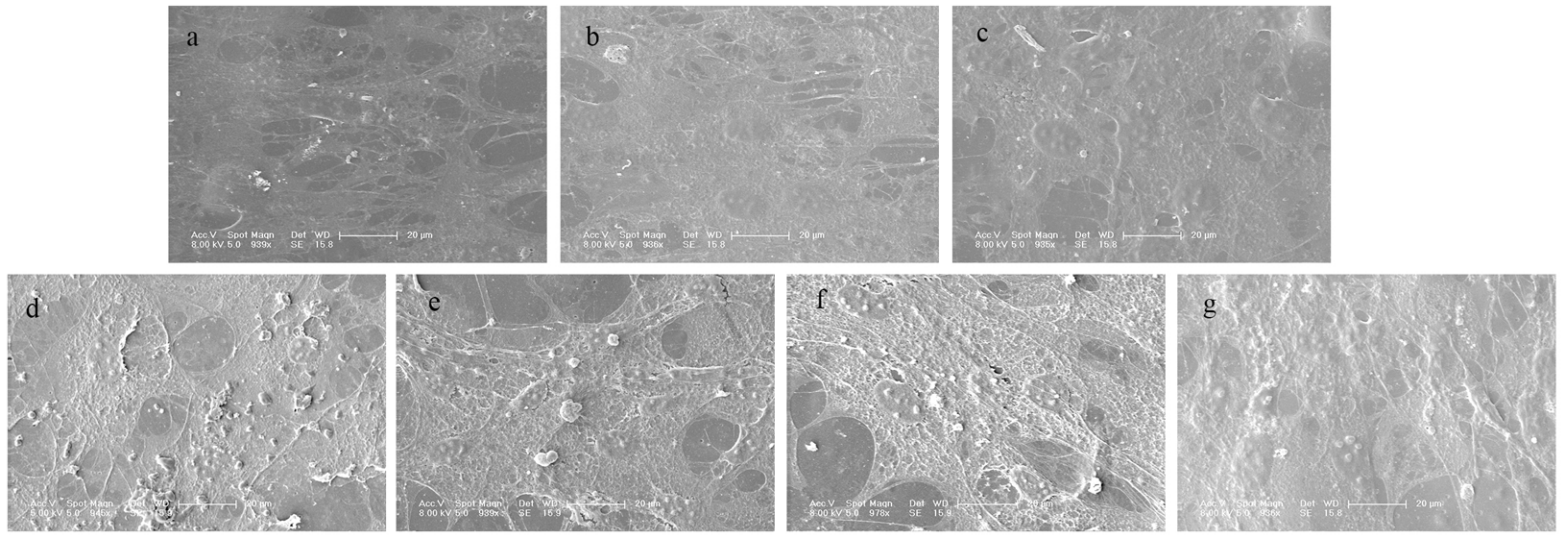
SEM images of MC3T3-E1 cells cultured for 21 days. (a) Control, (b) IGF (50 ng/ml), (c) IGF (100 ng/ml), (d) IGF (200 ng/ml), (e) ES+IGF (50 ng/ml), (f) ES+IGF (100 ng/ml), and (g) ES+IGF (200 ng/ml). All scale bar lengths are 20 μm.

Mineralization is the last phase of regeneration and is crucial for the final bone formation. To study the effect of ES and IGF on calcium uptake by MC3T3-E1 cells, the cells were stained with alizarin red to evaluate calcium mineralization quantitatively and qualitatively. As shown in Fig 8a, the total calcium content of the ES+IGF groups was greater than that of the IGF alone groups at the same dose, but a significant difference only occurred when the IGF concentration was 100 ng/ml and 200 ng/ml (p < 0.05, Fig 8). The optical images of the ARS staining showed little mineral deposition (red dots) in the IGF groups and the control group, while intense ARS staining was found in the ES+IGF (100 ng/ml) and ES+IGF (200 ng/ml) groups. The SEM images showed that the MC3T3-E1 cells growing in the ES+ IGF groups were more densely mineralized than those without ES. Compared to the control group, both the ES + IGF groups and the IGF alone groups had increased mineralized nodule formation, which is shown in Fig 9.

## Discussion

The treatment of large bone defects and nonunions are still the greatest challenges in orthopedic surgery. Growth factors and ES are widely used in bone regeneration because of their osteogenic activity. This study evaluates the function of IGF-1 and ES individually and together in osteogenesis.

To determine the most appropriate ES frequency for further experiments, we used an MTT assay to evaluate the cell proliferation. When the ES frequency ranged from 100 Hz-400 Hz, the cell proliferation activity was significantly higher than that in the control group without ES. The proliferation and adhesion of cells was the first step of osteogenesis, so we finally choose 200 Hz for further study since this frequency had the greatest effect on MC3T3-E1 proliferation. In our subsequent experiments, the results showed better cell adhesion with IGF-1 administered with ES than with the IGF-1 alone treatment, which indicated that ES can also affect the cell adhesion process.

The mechanism by which ES promotes bone cell proliferation and adhesion is still poorly understood. Studies showed that ES creates local electrical fields to influence extracellular matrix (ECM) proteins; these fields mediate the diffusion of proteins and their movement to sites on the surface of culture system[26]. Subsequently, the diffused proteins adsorbed on the surface to facilitate cell adhesion and spreading behaviors[27]. In addition, the electrical signal was considered to play a key role in cellular pathways involved in various cell activities, including proliferation and differentiation[28]. Therefore, we speculated that ES at 200 Hz can produce the optimal electrical fields and electrical signal. On the one hand, the electrical fields direct cells to move and adhere to the surface; on the other hand, the electrical signal may activate some cellular pathways related to cell proliferation.

The potential of IGF-1 to improve osteogenic proliferation and differentiation has been demonstrated, but the results are not consistent. Some studies indicated that IGF-1 is an important growth factor that can stimulate bone regeneration by enhancing osteoblast proliferation, bone matrix synthesis, osteoblastic gene expression, ALP activity and mineralization[15, 29–33]. However, some recent studies showed that IGF-1 alone does not improve osteogenic proliferation[23], ALP activity[34], osteogenic differentiation[22] and mineralization[35]. We speculated that IGF-1 has a dose-dependent effect on osteogenesis, and different IGF-1 doses may lead to different levels of osteoblastic proliferation or differentiation. In addition, the variety of cell types used for evaluating osteogenesis in different studies may also be responsible for the inconsistent results. Therefore, in this study, we aimed to evaluate the MC3T3-E1 osteogenesis induced by IGF-1 alone and by a combination treatment with ES, as well as the optimum IGF-1 dose.

Our result indicated that exogenous IGF-1 can stimulate the proliferation of MC3T3-E1 in vitro and that the 100 ng/ml concentration of IGF-1 promoted the most proliferation, which was in accordance with some previous studies[36, 37]. The addition of ES to the IGF-1 treatments led to a significantly higher increase, which suggests a synergistic relation between IGF-1 and ES that influences the proliferation of MC3T3-E1 cells. Osteoblast maturation can be divided into proliferation, differentiation and matrix mineralization[38]. We investigated the role of IGF-1 alone and in combination of ES in the differentiation and matrix mineralization phases by performing RT-PCR (RUNX2, OPN, and COL I) and by assaying the ALP activity and mineralization. We observed that IGF-1 at a concentration of 200 ng/mL significantly increased the RUNX2 and OPN levels; a change in ALP activity also occurred in MC3T3-E1 cultures at 7 and 14 days. However, the Col I gene expression increased most when the IGF-1 concentration was 100 ng/mL. RUNX2, OPN and Col I are three key osteoblast markers in the process of osteogenic differentiation. High RUNX2 expression was detected in preosteoblasts, immature osteoblasts, and early osteoblasts, which can be considered components of early differentiation[39]. OPN expression was observed at the middle/later stage of differentiation, and Col I represents the extracellular matrix during bone formation[40, 41]. ALP is an early marker of osteogenic differentiation, and ALP activity is closely related to osteogenic differentiation[42]. Our results demonstrated that IGF-1 can affect MC3T3-E1 cells at different stages of differentiation with a dose-dependent effect. IGF-1 at 50 ng/ml seemed to have no influence on the MC3T3-E1 cell differentiation phase. At 100 ng/ml, IGF-1 mainly enhanced bone collagen and matrix synthesis, as well as proliferation. At 200 ng/ml, IGF-1 significantly improved the early middle/later stage of differentiation. This finding indicated that IGF-1 at different concentrations affects all of the main functions in the osteogenesis process. When IGF-1 was combined with ES, no clear differences between the IGF-1+ES groups and the IGF-1 alone groups were found in the OPN gene expression; however, synergistic effects between IGF-1 and ES on the expression of the other two genes and the ALP activity were observed. This finding suggests that ES can actually enhance the IGF-1 effect on the early stage of MC3T3-E1 differentiation and matrix synthesis, whereas ES only slightly affected IGF-1 in the middle/later stage of differentiation. The result also revealed that ES might mainly activate cellular pathways of proliferation and the early stage of osteogenic differentiation.

Mineralization is the last phase of regeneration, which is crucial for final bone formation. The quantitative assessment of cell mineralization was performed by extracting Alizarin Red with 10% cetylpyridinium chloride (CPC), which was employed to determine calcium mineralization. From our result, it was also noteworthy that IGF-1 at a concentration of 100 ng/ml can distinctly promote calcium mineralization, as could its combination with ES. A previous study showed that mice lacking IGF-1R in osteoblasts failed to mineralize[43], indicating the key role of IGF-1 in the mineralization process. In consideration of 100 ng/ml IGF-1 having the strongest effect on proliferation and extracellular matrix synthesis, we speculated that these two main functions of IGF-1 might account for the higher mineralization and calcium deposition, which was also reported in other studies[15, 31, 44].

To summarize the osteogenic function of IGF-1, the positive effect of IGF-1 on the osteogenesis process did not follow a simple linear relationship; the appropriate dose should be determined based on the effect on the whole process of bone formation, not just proliferation or differentiation. From our data, 50 ng/ml IGF-1 only slightly affected the cell proliferation phase, which suggests that the low dose of IGF-1 had a very limited effect on osteogenesis. However, the higher dose of IGF-1 significantly promoted the whole phase of bone formation; 100 ng/ml focused on the proliferation, matrix synthesis and mineralization, while 200 ng/ml was inclined to affect cell differentiation. Since the function of IGF-1 in the osteogenesis process had a precise relation with IGF-1 dose, systemic application may not be suitable for IGF-1, particularly given its oncogenic effects when administered by systemic application[45]. Because of the synergistic effect between IGF-1 and ES on the whole osteogenic process, we suggest that a local combined treatment with IGF-1 and ES in the bone damage region has good prospects. A further study should explore a useful method to deliver IGF-1 and maintain the proper dose at the local bone damage region, which could maximize the clinical benefits of IGF-1.

Although our result indicated a synergistic effect between IGF-1 and ES on promoting osteogenic proliferation, differentiation and mineralization, the molecular mechanism is still unclear. IGF-1 binds and phosphorylates the membrane IGF-1 receptor (IGF-1R) to activate the RAS/MAP and PI3-kinase/Akt kinase pathways, leading to osteoblast differentiation and proliferation[38]. Therefore, we speculated that the quantitative and qualitative changes in IGF-1, the binding of IGF-1 and its receptor, and the interaction between IGF-1 and other cytokines may influence pathways and the final osteoblast activities. A previous study showed that ES can affect the signal transduction of the osteogenic process because of the structural change of osteogenesis-related cytokines and receptors[46]. ES can also stimulate the calcium-calmodulin pathway to upregulate bone morphogenetic proteins and other cytokines[6]. Furthermore, ES could promote BMP binding to BMPRs, which showed that ES can strengthen protein-receptor binding as well[47]. Analysis also showed that ES might affect the interaction between IGF-1 and its receptor, as well as the protein structure, thus exerting a synergistic effect with IGF-1. In a future study, we will verify the hypothetical molecular mechanisms of ES synergistically facilitating the effect of IGF-1 on the whole osteogenesis process.

### Conclusion

We have demonstrated that IGF-1 and ES individually and collectively were able to promote cell viability, the expression of osteogenic genes, ALP activity and extracellular calcium deposition in a clear dose-dependent manner. These findings highlight the synergistic effect of IGF-1 and ES in promoting the proliferation, differentiation and mineralization of MC3T3-E1 cells. The present study is the first demonstration of the interactive effects of IGF-1 and ES on osteogenic development, which suggest that it would be more effective to combine the proper dose of IGF-1 with ES to promote local bone damage repair and regeneration.

## Supporting information

S1 Data Minimal data set. Individual data points for graphs and figures in this research.
(XLSX)

## References

[1] Cook JJ, Summers NJ, Cook EA. Healing in the new millennium: bone stimulators: an overview of where we’ve been and where we may be heading. Clinics in podiatric medicine and surgery. 2015;32(1):45–59. doi: 10.1016/j.cpm.2014.09.003 . 25440417 10.1016/j.cpm.2014.09.003

[2] Ciombor DM, Aaron RK. The role of electrical stimulation in bone repair. Foot and ankle clinics. 2005;10(4):579–93, vii. doi: 10.1016/j.fcl.2005.06.006 . 16297820 10.1016/j.fcl.2005.06.006

[3] Ebrahim S, Mollon B, Bance S, Busse JW, Bhandari M. Low-intensity pulsed ultrasonography versus electrical stimulation for fracture healing: a systematic review and network meta-analysis. Canadian journal of surgery Journal canadien de chirurgie. 2014;57(3):E105–18. doi: 10.1503/cjs.010113 . 24869616 10.1503/cjs.010113 PMC4035413

[4] Goldstein C, Sprague S, Petrisor BA. Electrical stimulation for fracture healing: current evidence. Journal of orthopaedic trauma. 2010;24 Suppl 1:S62–5. doi: 10.1097/BOT.0b013e3181cdde1b . 20182239 10.1097/BOT.0b013e3181cdde1b

[5] Haddad JB, Obolensky AG, Shinnick P. The biologic effects and the therapeutic mechanism of action of electric and electromagnetic field stimulation on bone and cartilage: new findings and a review of earlier work. Journal of alternative and complementary medicine. 2007;13(5):485–90. doi: 10.1089/acm.2007.5270 . 17604552 10.1089/acm.2007.5270

[6] Aleem IS, Aleem I, Evaniew N, Busse JW, Yaszemski M, Agarwal A, et al Efficacy of Electrical Stimulators for Bone Healing: A Meta-Analysis of Randomized Sham-Controlled Trials. Scientific reports. 2016;6:31724 doi: 10.1038/srep31724 . 27539550 10.1038/srep31724 PMC4990885

[7] Bodamyali T, Bhatt B, Hughes FJ, Winrow VR, Kanczler JM, Simon B, et al Pulsed electromagnetic fields simultaneously induce osteogenesis and upregulate transcription of bone morphogenetic proteins 2 and 4 in rat osteoblasts in vitro. Biochemical and biophysical research communications. 1998;250(2):458–61. doi: 10.1006/bbrc.1998.9243 . 9753652 10.1006/bbrc.1998.9243

[8] Griffin M, Iqbal SA, Sebastian A, Colthurst J, Bayat A. Degenerate wave and capacitive coupling increase human MSC invasion and proliferation while reducing cytotoxicity in an in vitro wound healing model. PloS one. 2011;6(8):e23404 doi: 10.1371/journal.pone.0023404 . 21858102 10.1371/journal.pone.0023404 PMC3156742

[9] Mobini S, Leppik L, Thottakkattumana Parameswaran V, Barker JH. In vitro effect of direct current electrical stimulation on rat mesenchymal stem cells. PeerJ. 2017;5:e2821 doi: 10.7717/peerj.2821 . 28097053 10.7717/peerj.2821 PMC5237370

[10] Wiesmann H, Hartig M, Stratmann U, Meyer U, Joos U. Electrical stimulation influences mineral formation of osteoblast-like cells in vitro. Biochimica et biophysica acta. 2001;1538(1):28–37. . 11341980 10.1016/s0167-4889(00)00135-x

[11] Chisalita SI, Chong LT, Wajda M, Adolfsson L, Woisetschlager M, Spangeus A. Association of Insulin-like Growth Factor-1, Bone Mass and Inflammation to Low-energy Distal Radius Fractures and Fracture Healing in Elderly Women Attending Emergency Care. Orthopaedic surgery. 2017;9(4):380–5. doi: 10.1111/os.12358 . 29178313 10.1111/os.12358 PMC6584470

[12] Di Luca A, Klein-Gunnewiek M, Vancso JG, van Blitterswijk CA, Benetti EM, Moroni L. Covalent Binding of Bone Morphogenetic Protein-2 and Transforming Growth Factor-beta3 to 3D Plotted Scaffolds for Osteochondral Tissue Regeneration. Biotechnology journal. 2017;12(12). doi: 10.1002/biot.201700072 . 28865136 10.1002/biot.201700072

[13] Naskar D, Ghosh AK, Mandal M, Das P, Nandi SK, Kundu SC. Dual growth factor loaded nonmulberry silk fibroin/carbon nanofiber composite 3D scaffolds for in vitro and in vivo bone regeneration. Biomaterials. 2017;136:67–85. doi: 10.1016/j.biomaterials.2017.05.014 . 28521202 10.1016/j.biomaterials.2017.05.014

[14] Ren Q, Cai M, Zhang K, Ren W, Su Z, Yang T, et al Effects of bone morphogenetic protein-2 (BMP-2) and vascular endothelial growth factor (VEGF) release from polylactide-poly (ethylene glycol)-polylactide (PELA) microcapsule-based scaffolds on bone. Brazilian journal of medical and biological research = Revista brasileira de pesquisas medicas e biologicas. 2017;51(2):e6520 doi: 10.1590/1414-431X20176520 . 29211249 10.1590/1414-431X20176520 PMC5711005

[15] Wang S, Mu J, Fan Z, Yu Y, Yan M, Lei G, et al Insulin-like growth factor 1 can promote the osteogenic differentiation and osteogenesis of stem cells from apical papilla. Stem cell research. 2012;8(3):346–56. doi: 10.1016/j.scr.2011.12.005 . 22286010 10.1016/j.scr.2011.12.005

[16] Chen L, Zou X, Zhang RX, Pi CJ, Wu N, Yin LJ, et al IGF1 potentiates BMP9-induced osteogenic differentiation in mesenchymal stem cells through the enhancement of BMP/Smad signaling. BMB reports. 2016;49(2):122–7. doi: 10.5483/BMBRep.2016.49.2.228 . 26645636 10.5483/BMBRep.2016.49.2.228 PMC4915116

[17] Choi GH, Lee HJ, Lee SC. Titanium-adhesive polymer nanoparticles as a surface-releasing system of dual osteogenic growth factors. Macromolecular bioscience. 2014;14(4):496–507. doi: 10.1002/mabi.201300368 . 24227631 10.1002/mabi.201300368

[18] Kim S, Kang Y, Krueger CA, Sen M, Holcomb JB, Chen D, et al Sequential delivery of BMP-2 and IGF-1 using a chitosan gel with gelatin microspheres enhances early osteoblastic differentiation. Acta biomaterialia. 2012;8(5):1768–77. doi: 10.1016/j.actbio.2012.01.009 . 22293583 10.1016/j.actbio.2012.01.009 PMC3314097

[19] Tahimic CG, Wang Y, Bikle DD. Anabolic effects of IGF-1 signaling on the skeleton. Frontiers in endocrinology. 2013;4:6 doi: 10.3389/fendo.2013.00006 . 23382729 10.3389/fendo.2013.00006 PMC3563099

[20] Jiang J, Lichtler AC, Gronowicz GA, Adams DJ, Clark SH, Rosen CJ, et al Transgenic mice with osteoblast-targeted insulin-like growth factor-I show increased bone remodeling. Bone. 2006;39(3):494–504. doi: 10.1016/j.bone.2006.02.068 . 16644298 10.1016/j.bone.2006.02.068

[21] Zhao G, Monier-Faugere MC, Langub MC, Geng Z, Nakayama T, Pike JW, et al Targeted overexpression of insulin-like growth factor I to osteoblasts of transgenic mice: increased trabecular bone volume without increased osteoblast proliferation. Endocrinology. 2000;141(7):2674–82. doi: 10.1210/endo.141.7.7585 . 10875273 10.1210/endo.141.7.7585

[22] Walsh S, Jefferiss CM, Stewart K, Beresford JN. IGF-I does not affect the proliferation or early osteogenic differentiation of human marrow stromal cells. Bone. 2003;33(1):80–9. . 12919702 10.1016/s8756-3282(03)00165-0

[23] Shu X, Feng J, Feng J, Huang X, Li L, Shi Q. Combined delivery of bone morphogenetic protein-2 and insulin-like growth factor-1 from nano-poly (gamma-glutamic acid)/beta-tricalcium phosphate-based calcium phosphate cement and its effect on bone regeneration in vitro. Journal of biomaterials applications. 2017;32(5):547–60. doi: 10.1177/0885328217737654 . 29113568 10.1177/0885328217737654

[24] Duruel T, Cakmak AS, Akman A, Nohutcu RM, Gumusderelioglu M. Sequential IGF-1 and BMP-6 releasing chitosan/alginate/PLGA hybrid scaffolds for periodontal regeneration. International journal of biological macromolecules. 2017;104(Pt A):232–41. doi: 10.1016/j.ijbiomac.2017.06.029 . 28601648 10.1016/j.ijbiomac.2017.06.029

[25] Park H, Larson BL, Kolewe ME, Vunjak-Novakovic G, Freed LE. Biomimetic scaffold combined with electrical stimulation and growth factor promotes tissue engineered cardiac development. Experimental cell research. 2014;321(2):297–306. doi: 10.1016/j.yexcr.2013.11.005 . 24240126 10.1016/j.yexcr.2013.11.005 PMC3946629

[26] Kotwal A, Schmidt CE. Electrical stimulation alters protein adsorption and nerve cell interactions with electrically conducting biomaterials. Biomaterials. 2001;22(10):1055–64. . 11352099 10.1016/s0142-9612(00)00344-6

[27] Parsons JT, Horwitz AR, Schwartz MA. Cell adhesion: integrating cytoskeletal dynamics and cellular tension. Nature reviews Molecular cell biology. 2010;11(9):633–43. doi: 10.1038/nrm2957 . 20729930 10.1038/nrm2957 PMC2992881

[28] Levin M. Bioelectric mechanisms in regeneration: Unique aspects and future perspectives. Seminars in cell & developmental biology. 2009;20(5):543–56. doi: 10.1016/j.semcdb.2009.04.013 . 19406249 10.1016/j.semcdb.2009.04.013 PMC2706303

[29] Eriksen EF, Kassem M, Langdahl B. Growth hormone, insulin-like growth factors and bone remodelling. European journal of clinical investigation. 1996;26(7):525–34. . 8864413 10.1046/j.1365-2362.1996.00292.x

[30] Yao W, Zhong J, Yu J, Warner T, Bozic T, Ye P, et al IGF-I improved bone mineral density and body composition of weaver mutant mice. Growth hormone & IGF research: official journal of the Growth Hormone Research Society and the International IGF Research Society. 2008;18(6):517–25. doi: 10.1016/j.ghir.2008.04.006 . 18550407 10.1016/j.ghir.2008.04.006 PMC2633297

[31] Zhang W, Shen X, Wan C, Zhao Q, Zhang L, Zhou Q, et al Effects of insulin and insulin-like growth factor 1 on osteoblast proliferation and differentiation: differential signalling via Akt and ERK. Cell biochemistry and function. 2012;30(4):297–302. doi: 10.1002/cbf.2801 . 22249904 10.1002/cbf.2801

[32] Guerra-Menendez L, Sadaba MC, Puche JE, Lavandera JL, de Castro LF, de Gortazar AR, et al IGF-I increases markers of osteoblastic activity and reduces bone resorption via osteoprotegerin and RANK-ligand. Journal of translational medicine. 2013;11:271 doi: 10.1186/1479-5876-11-271 . 24161214 10.1186/1479-5876-11-271 PMC4231608

[33] Feng X, Huang D, Lu X, Feng G, Xing J, Lu J, et al Insulin-like growth factor 1 can promote proliferation and osteogenic differentiation of human dental pulp stem cells via mTOR pathway. Development, growth & differentiation. 2014;56(9):615–24. doi: 10.1111/dgd.12179 . 25388971 10.1111/dgd.12179

[34] Rico-Llanos GA, Becerra J, Visser R. Insulin-like growth factor-1 (IGF-1) enhances the osteogenic activity of bone morphogenetic protein-6 (BMP-6) in vitro and in vivo, and together have a stronger osteogenic effect than when IGF-1 is combined with BMP-2. Journal of biomedical materials research Part A. 2017;105(7):1867–75. doi: 10.1002/jbm.a.36051 . 28256809 10.1002/jbm.a.36051

[35] Doorn J, Roberts SJ, Hilderink J, Groen N, van Apeldoorn A, van Blitterswijk C, et al Insulin-like growth factor-I enhances proliferation and differentiation of human mesenchymal stromal cells in vitro. Tissue engineering Part A. 2013;19(15–16):1817–28. doi: 10.1089/ten.TEA.2012.0522 . 23530894 10.1089/ten.TEA.2012.0522

[36] Jeong WK, Park SW, Im GI. Growth factors reduce the suppression of proliferation and osteogenic differentiation by titanium particles on MSCs. Journal of biomedical materials research Part A. 2008;86(4):1137–44. doi: 10.1002/jbm.a.32068 . 18442110 10.1002/jbm.a.32068

[37] Chen FM, Chen R, Wang XJ, Sun HH, Wu ZF. In vitro cellular responses to scaffolds containing two microencapulated growth factors. Biomaterials. 2009;30(28):5215–24. doi: 10.1016/j.biomaterials.2009.06.009 . 19560814 10.1016/j.biomaterials.2009.06.009

[38] Valenti MT, Dalle Carbonare L, Mottes M. Osteogenic Differentiation in Healthy and Pathological Conditions. International journal of molecular sciences. 2016;18(1). doi: 10.3390/ijms18010041 . 28035992 10.3390/ijms18010041 PMC5297676

[39] Komori T. Regulation of bone development and extracellular matrix protein genes by RUNX2. Cell and tissue research. 2010;339(1):189–95. doi: 10.1007/s00441-009-0832-8 . 19649655 10.1007/s00441-009-0832-8

[40] Blanquer A, Musilkova J, Barrios L, Ibanez E, Vandrovcova M, Pellicer E, et al Cytocompatibility assessment of Ti-Zr-Pd-Si-(Nb) alloys with low Young’s modulus, increased hardness, and enhanced osteoblast differentiation for biomedical applications. Journal of biomedical materials research Part B, Applied biomaterials. 2017 doi: 10.1002/jbm.b.33892 . 28390183 10.1002/jbm.b.33892

[41] Yu M, Wang L, Ba P, Li L, Sun L, Duan X, et al Osteoblast Progenitors Enhance Osteogenic Differentiation of Periodontal Ligament Stem Cells. Journal of periodontology. 2017;88(10):e159–e68. doi: 10.1902/jop.2017.170016 . 28517970 10.1902/jop.2017.170016

[42] Wennberg C, Hessle L, Lundberg P, Mauro S, Narisawa S, Lerner UH, et al Functional characterization of osteoblasts and osteoclasts from alkaline phosphatase knockout mice. Journal of bone and mineral research: the official journal of the American Society for Bone and Mineral Research. 2000;15(10):1879–88. doi: 10.1359/jbmr.2000.15.10.1879 . 11028439 10.1359/jbmr.2000.15.10.1879

[43] Zhang M, Xuan S, Bouxsein ML, von Stechow D, Akeno N, Faugere MC, et al Osteoblast-specific knockout of the insulin-like growth factor (IGF) receptor gene reveals an essential role of IGF signaling in bone matrix mineralization. The Journal of biological chemistry. 2002;277(46):44005–12. doi: 10.1074/jbc.M208265200 . 12215457 10.1074/jbc.M208265200

[44] Thomas T, Gori F, Spelsberg TC, Khosla S, Riggs BL, Conover CA. Response of bipotential human marrow stromal cells to insulin-like growth factors: effect on binding protein production, proliferation, and commitment to osteoblasts and adipocytes. Endocrinology. 1999;140(11):5036–44. doi: 10.1210/endo.140.11.7128 . 10537129 10.1210/endo.140.11.7128

[45] Sugumar A, Liu YC, Xia Q, Koh YS, Matsuo K. Insulin-like growth factor (IGF)-I and IGF-binding protein 3 and the risk of premenopausal breast cancer: a meta-analysis of literature. International journal of cancer. 2004;111(2):293–7. doi: 10.1002/ijc.20253 . 15197785 10.1002/ijc.20253

[46] Azimi O, Emami Z, Salari H, Chamani J. Probing the interaction of human serum albumin with norfloxacin in the presence of high-frequency electromagnetic fields: fluorescence spectroscopy and circular dichroism investigations. Molecules. 2011;16(12):9792–818. doi: 10.3390/molecules16129792 . 22117170 10.3390/molecules16129792 PMC6264156

[47] Wang Y, Cui H, Wu Z, Wu N, Wang Z, Chen X, et al Modulation of Osteogenesis in MC3T3-E1 Cells by Different Frequency Electrical Stimulation. PloS one. 2016;11(5):e0154924 doi: 10.1371/journal.pone.0154924 . 27149625 10.1371/journal.pone.0154924 PMC4858221

